# Nanomedicines for cancer therapy: state-of-the-art and limitations to pre-clinical studies that hinder future developments

**DOI:** 10.3389/fchem.2014.00069

**Published:** 2014-08-25

**Authors:** Charlene M. Dawidczyk, Luisa M. Russell, Peter C. Searson

**Affiliations:** ^1^Institute for Nanobiotechnology, Johns Hopkins UniversityBaltimore, MD, USA; ^2^Johns Hopkins Center of Cancer Nanotechnology Excellence, Johns Hopkins UniversityBaltimore, MD, USA; ^3^Department of Materials Science and Engineering, Johns Hopkins UniversityBaltimore, MD, USA

**Keywords:** drug delivery systems, nanoparticles, targeted therapy, pharmacokinetics, tumor accumulation

## Abstract

The ability to efficiently deliver a drug or gene to a tumor site is dependent on a wide range of factors including circulation time, interactions with the mononuclear phagocyte system, extravasation from circulation at the tumor site, targeting strategy, release from the delivery vehicle, and uptake in cancer cells. Nanotechnology provides the possibility of creating delivery systems where the design constraints are decoupled, allowing new approaches for reducing the unwanted side effects of systemic delivery, increasing tumor accumulation, and improving efficacy. The physico-chemical properties of nanoparticle-based delivery platforms introduce additional complexity associated with pharmacokinetics, tumor accumulation, and biodistribution. To assess the impact of nanoparticle-based delivery systems, we first review the design strategies and pharmacokinetics of FDA-approved nanomedicines. Next we review nanomedicines under development, summarizing the range of nanoparticle platforms, strategies for targeting, and pharmacokinetics. We show how the lack of uniformity in preclinical trials prevents systematic comparison and hence limits advances in the field.

## Introduction

Drug therapy often involves the use of small molecules such as alkylating agents (e.g., busulfan), anti-metabolites (e.g., gemcitabine), anti-microtubule agents (e.g., paclitaxel, vincristine), topoisomerase inhibitors (e.g., topotecan), and cytotoxic inhibitors (e.g., doxorubicin). These cytotoxic molecules kill highly proliferative cancer cells, but also other proliferative cells in bone marrow, the gastrointestinal (GI) tract, and hair follicles, leading to common side effects such as compromised immune system, inflammation and ulceration of the GI tract, and hair loss. Nanotechnology provides the possibility of creating delivery systems where the design constraints are decoupled, allowing new approaches for reducing the unwanted side effects of systemic delivery, increasing tumor accumulation, and improving efficacy.

The development of safe and efficient delivery systems is also important for advances in human gene therapy (Pack et al., 2005; Jones et al., 2013). A delivery system must transport a gene with high efficiency to target cells, with minimal toxicity and immune response. The main challenges for gene delivery are protecting the genetic material from degradation in circulation, avoiding degradation by enzymes in endosomes in the target cell, and escaping from endosomes to reach the nucleus or target compartment (Mintzer and Simanek, 2009; Zhang et al., 2012).

Key properties for drug and gene delivery systems are biocompatibility, stability in circulation, and increasing the fraction of the dose accumulating in the tumor. Drug toxicity can be reduced by encapsulating the free drug (e.g., liposomes) or by locally activating a pro-drug. Stability in circulation can be improved by developing strategies to minimize protein binding and evade the immune system. The efficiency of accumulation at a tumor site can be improved by active targeting of the delivery system or by increasing extravasation by the enhanced permeation and retention (EPR) effect.

The FDA-approved nanomedicines in clinical use have demonstrated the potential for increasing bioavailability, enhancing drug solubility, active targeting, and high drug loading (Dawidczyk et al., 2014). However, there remain many challenges in exploiting advances in nanotechnology and bioengineering to develop systems that will have significant impact on patient survival rates. The development of delivery systems remains largely empirical and the lack of standardization of pre-clinical studies is a barrier to establishing design rules for nanomedicines. While studies of complex systems with combined reporting/sensing functions along with drug or gene delivery may ultimately improve diagnosis and treatment, there are many fundamental issues that need to be addressed to establish the relationship between physico-chemical properties, pharmacokinetics, biodistribution, and survival rates.

Tumor uptake is modulated by the EPR effect (Jain and Stylianopoulos, 2010; Fang et al., 2011; Torchilin, 2011) and hence increasing the circulation time generally increases tumor accumulation. A common approach for increasing circulation time is to provide a surface coating of polyethylene glycol (PEG). Whilst PEG coating increases circulation time and hence can increase tumor accumulation, it also inhibits uptake by tumor cells (Barenholz, 2012). Furthermore, PEG coating slows but does not prevent adsorption of opsonins that promote uptake by macrophages of the liver and spleen. Targeting molecules such as immunoglobulin-G (IgG) antibodies are opsonins and hence promote clearance by the mononuclear phagocyte system (MPS) (Walkey and Chan, 2012). The conjugation of folate to liposomes significantly increases their uptake by tumor-associated macrophages (Turk et al., 2004).

### FDA-approved nanomedicines

There are currently six FDA-approved nanomedicines (Table 1): brentuximab vedotin and Trastuzumab emtansine, Doxil, DaunoXome, Marqibo, and Abraxane (Dawidczyk et al., 2014). Brentuximab vedotin and Trastuzumab emtansine are antibody-drug conjugates (ADCs), conceptually one of the simplest nanomedicines with an anticancer drug conjugated to a targeting molecule. Brentuximab targets the protein CD30, a glycosylated phosphoprotein expressed by B cells, including B-cell lymphomas, some leukemias, and melanoma cancer stem cells (Mullard, 2013; Sassoon and Blanc, 2013; Sievers and Senter, 2013). Trastuzumab targets the human epidermal growth factor receptor 2 (HER2) overexpressed in HER2 positive breast cancer (Lu et al., 2012; Verma et al., 2012). Monomethyl auristan E (MMAE) (Brentuximab vedotin) and mertansine (Trastuzumab emtansine) are too toxic to be used alone and hence coupling to a targeting antibody reduces toxic side effects. Several drug molecules are conjugated to each antibody via a valine-citrulline cleavable linker (Brentuximab vedotin) or covalent linkage (Trastuzumab emtansine) that is enzymatically degraded in endosomes following uptake. The small number of FDA-approved ADCs highlights the difficulty in translating relatively simple nanomedicines to the clinic.

**Table 1:** **Summary of FDA-approved nanomedicines**.

**Platform**	**Class**	**Drug**	**d (nm)**	**Drug/carrier ratio**	**Key design feature(s)**	**Problem addressed**
Brentuximab vedotin	ADC	Monomethyl auristan E	~10	≤8	Valine-citrulline linker cleaved by cathepsin in endosomes	Monomethyl auristan E (MMAE) is too toxic to be used alone
Trastuzumab emtansine	ADC	Mertansine	~10	≤8	Non-cleavable linker; release of drug by proteolytic degradation of antibody in endosomes	Mertansine is too toxic to be used alone
Doxil	Liposome	Doxorubicin	100	10,000–15,000	Lipid encapsulation for high drug/carrier ratio, polyethylene glycol coating to evade MPS, crystallization of drug in liposome minimizes escape during circulation	Drug toxicity and adverse cardiac side effects
DaunoXome	Liposome	Daunorubicin	50	~10,000	No polyethylene glycol coating, targeted by MPS resulting in slow release into circulation	Drug toxicity and adverse cardiac side effects
Marqibo	Liposome	Vincristine	100	~10,000	No polyethylene glycol coating, targeted by MPS resulting in slow release into circulation	Drug toxicity and adverse side effects
Abraxane	Protein carrier	Paclitaxel	130	>10,000	Non-specific binding of paclitaxel to albumin	Overcomes very low solubility of paclitaxel

Doxil, DaunoXome, and Marqibo are liposomal nanomedicines. Doxil is a pegylated liposome about 100 nm in diameter and encapsulating about 10,000 doxorubicin molecules (Barenholz, 2012). Encapsulation minimizes side effects, such as cardiotoxicity, associated with high doses of free doxorubicin. The concentration of doxorubicin in the liposomes is greater than the solubility limit and hence most of the drug is in the solid phase (Barenholz, 2012). The incorporation of cholesterol increases the bilayer cohesiveness and reduces leakage. These features minimize osmotic effects and contribute to stability, with more than 98% of the circulating drug remaining inside liposomes (Lasic et al., 1992; Gabizon et al., 1994, 2003). The polyethylene glycol coating is designed to give a long circulation half time and thereby increase tumor accumulation by the EPR effect (Immordino et al., 2006; Vllasaliu et al., 2014). While the mechanisms of uptake and release are not known, evidence suggests that the liposomes are taken up by endocytosis (Seynhaeve et al., 2013).

DaunoXome (Gill et al., 1996; Bellott et al., 2001; Lowis et al., 2006) and Marqibo (Bedikian et al., 2011; Silverman and Deitcher, 2013) are liposomal formulations of daunorubicin and vincristine, respectively. In contrast to Doxil, the design strategy for DaunoXome and Marqibo is to promote uptake by the MPS, providing a reservoir from which the free drug can enter circulation, similar to a slow infusion. This is achieved by not including pegylated lipids in the liposomes (Gill et al., 1996; Bellott et al., 2001; Silverman and Deitcher, 2013). DaunoXome is about 50 nm in diameter (Gill et al., 1996), and Marqibo is about 100 nm in diameter (Silverman and Deitcher, 2013).

Abraxane, or nab-paclitaxel (nanoparticle albumin bound), is lyophilized human serum albumin non-specifically bound to paclitaxel (Miele et al., 2009). Paclitaxel has very low solubility and is administered with the toxic non-ionic solvent Cremophor, which can lead to a wide range of allergic reactions. On injection, Abraxane particles dissociate into smaller albumin-paclitaxel complexes or unbound paclitaxel (Yardley, 2013). Since albumin is abundant in circulation, Abraxane provides a reservoir of a very low solubility drug in a non-toxic platform. The particles are about 130 nm in diameter and contain about 10,000 paclitaxel molecules (Miele et al., 2009).

The pharmacokinetics of these nanomedicines reflects their design (Table 2). Brentuximab vedotin and Trastuzumab emtansine both have moderate areas under the curve (AUCs), relatively low clearance, and long elimination half-times of 3–4 days (Younes et al., 2010; Lorusso et al., 2011; Girish et al., 2012; Lu et al., 2012; Bradley et al., 2013). Doxil has high AUC, low clearance rate, small distribution volume, and a long elimination half-time (Barenholz, 2012). These features are largely due to the polyethylene glycol coating that provides extended evasion of the MPS and minimizes distribution into peripheral tissues (Gabizon et al., 1994; Hubert et al., 2000; Lyass et al., 2000; Hong and Tseng, 2001; Hamilton et al., 2002). DaunoXome (Gill et al., 1996; Bellott et al., 2001; Lowis et al., 2006) and Marqibo (Bedikian et al., 2011; Silverman and Deitcher, 2013) have clearance rates about an order of magnitude larger than for the ADCs and Doxil, low distribution volumes, and short elimination half-times on the order of 10 h. The larger AUC associated with DaunoXome is related to the larger dose range compared to Marqibo. Abraxane has a fast clearance rate, about two orders of magnitudes larger than DaunoXome and Marqibo, large distribution volume, and elimination half-time similar to DaunoXome and Marqibo (Sparreboom et al., 2005; Ando et al., 2012). The pharmacokinetics for Abraxane are similar to free paclitaxel and the other free drugs: low AUC, high clearance rate, high distribution volume, and short elimination half-time.

**Table 2:** **Summary of pharmacokinetics for FDA-approved nanomedicines and corresponding free drugs from human clinical trials**.

**Drug**	**Dose mg^2^/m**	**AUC (mg h/L)**	**CL (L/h)**	**V_d_(L)**	**t_1/2_(h)**	**References**
Brentuximab vedotin	90–110	3.2–4.9	0.071–0.075	8.2–10.2	106–144	Younes et al., 2010; Bradley et al., 2013
Trastuzumab emtansine	10–160	0.6–28	0.023–0.070	1.7–3.5	31–98	Lorusso et al., 2011; Girish et al., 2012; Lu et al., 2012
Doxil	25–80	600–4900	0.023–0.045	2.1–6.4	42–90	Gabizon et al., 1994; Hubert et al., 2000; Lyass et al., 2000; Hong and Tseng, 2001; Hamilton et al., 2002
DaunoXome	10–190	17–1700	0.40–0.94	2.9–4.1	2.8–8.3	Gill et al., 1996; Bellott et al., 2001; Lowis et al., 2006
Marqibo	2.0–2.25	5–15	0.36–0.38	2.6–2.9	9.6–12	Bedikian et al., 2011; Silverman and Deitcher, 2013
Abraxane	150–300	4–10	31–67	900–1700	11–26	Sparreboom et al., 2005; Ando et al., 2012
Doxorubicin	15–72	0.5–3.8	25–72	250–1800	9–29	Erttmann et al., 1988; Jacquet et al., 1990; Piscitelli et al., 1993; Gabizon et al., 1994
Daunorubicin	40–120	1–19	110–150	200–450	9–24	Bellott et al., 2001; Krogh-Madsen et al., 2012
Paclitaxel	170–330	6–40	15–50	160–530	7.2–7.6	Sparreboom et al., 2005

In most cases, data represent the range of mean or median values for obtained from different doses. For unit conversion we used an average body surface area of 1.7 m^2^, an average body weight of 60 kg, and a blood volume of 5 L.

Overall it is evident that antibody drug conjugates or liposomes with a pegylated surface have long elimination half-times, typically of 3–4 days. Increasing elimination half-times is expected to increase tumor accumulation via the EPR effect. However, increased tumor accumulation does not necessarily imply improved efficacy since processes such as transport, uptake, drug release, and delivery to the appropriate cellular compartment are all downstream of extravasation by the EPR effect.

## Nanoparticle platforms, targeting moieties

### Nanoparticle platforms

The development of a broad range of nanoparticle platforms with the ability to tune size, composition, and functionality has provided a significant resource for nanomedicine (Table 3) (Niemeyer, 2001; Duncan, 2006; Cho et al., 2008; Greco and Vicent, 2009; Yu et al., 2013). Nanoparticle platforms can be broadly categorized as organic, inorganic, and hybrid.

**Table 3:** **Summary of nanoparticle platforms for nanomedicine**.

	**Particle type**	**Composition/Structure**	**Properties**	**Applications**
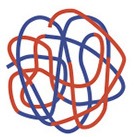	Polymer	e.g., PLGA, glycerol, chitosan, DNA; monomers, copolymers, hydrogels	Some biodegradable	Drug delivery; passive release (diffusion), controlled release (triggered)
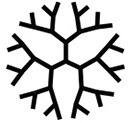	Dendrimer	PAMAM, etc.	Low polydispersity, cargo, biocompatible	Drug delivery
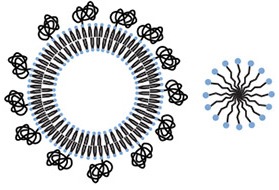	Lipid	Liposomes, micelles	Can carry hydrophobic cargo, biocompatible, typically 50–500 nm	Drug delivery
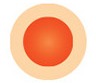	Quantum dots	CdSe, CulnSe, CdTe, etc.	Broad excitation, no photobleaching, tunable emission, typically 5–100 nm	Optical imaging
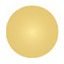	Gold	Spheres, rods, or shells	Biocompatibility, typically 5–100 nm	Hyperthermia therapy, drug delivery
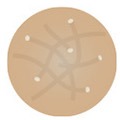	Silica	Spheres, shells, mesoporous	Biocompatibility	Contrast agents, drug delivery (encapsulation)
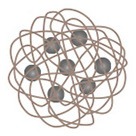	Magnetic	Iron oxide or cobalt-based; spheres, aggregates in dextran or silica	Superparamagnetic, ferromagnetic (small remanence to minimize aggregation), superferromagnetic (~10 nm), paramagnetic	Contrast agents (MRI), hyperthermia therapy
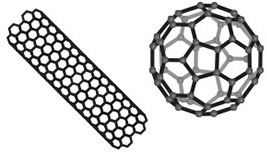	Carbon-based	Carbon nanotubes, buckyballs, graphene	Biocompatible	Drug delivery

Organic nanoparticles have been widely explored for decades, yielding a large variety of materials, formulations, imaging modalities, cargo, and targets for cancer therapy. Organic polymer systems include synthetic polymers [e.g., polyethyleneimine (PEI), polyethylene glycol] (Knop et al., 2010; Nicolas et al., 2013), synthetic hydrogels (e.g., polyacrylamide) (Ando et al., 2012; Liechty and Peppas, 2012), natural polymers (e.g., chitosan, hyaluronic acid, alginate, gelatin) (Ando et al., 2012) and hydrolytically or enzymatically degradable polymers (e.g., collagen, polylactic acid, polycaprolactone) (Balogh et al., 2007). Combinations of components and/or monomer units and incorporation of other building blocks such as DNA contribute to the flexibility of polymer-based nanoparticle platforms. These systems can be passively loaded with a cargo, or a cargo can be incorporated to allow triggered release (Davis et al., 2008). Particles such as block copolymers, liposomes, and dendrimers can provide a reservoir for large amounts of cargo. Block copolymers combine the attributes of two or more monomer units allowing further functionality (Duncan, 2006; Greco and Vicent, 2009). Lipid-based nanoparticles include micelles, liposomes, or water oil emulsions. Dendrimers are hyperbranched synthetic polymers for which biodistribution, size, and multifunctionality can be tuned with a very low degree of polydispersity (Cho et al., 2008). Proteins (e.g., albumin) (Fuchs and Coester, 2010) and viruses (Steinmetz, 2010) have also been extensively studied for drug and gene delivery.

Inorganic nanoparticles provide advantages in function and properties not possible with organic nanoparticle platforms, although this is often at the expense of biocompatibility. Examples of materials include semiconductors (quantum dots) (Gao et al., 2004; Medintz et al., 2005; Michalet et al., 2005; Park et al., 2011; Chen et al., 2012a; Petryayeva et al., 2013), silica (Vanblaaderen and Vrij, 1992; Giri et al., 2005, 2007; Burns et al., 2006), gold (Boisselier and Astruc, 2009; Arvizo et al., 2010), magnetic materials (Arruebo et al., 2007; Banerjee et al., 2010; Haun et al., 2010), and carbon-based materials (Prato et al., 2008; Jain, 2012). Semiconductor nanoparticles, or quantum dots, have a narrow and tunable emission spectrum, a broad excitation spectrum, and do not photobleach. These characteristics are attractive for optical imaging, however, many quantum dots are synthesized from heavy metal elements and hence toxicity is a concern. Silicon dioxide (silica), the most widely used oxide, is a versatile material that is relatively inert. Silica can be used to encapsulate other materials or cargoes and the surface can be conjugated using silane chemistry. Silica can be synthesized with nanometer scale pores (mesoporous silica) that can be used to hold other cargoes. Of the metallic materials, gold is widely used for biological applications as it is easy to synthesize, can be functionalized using thiol chemistry, and is relatively inert. Many of the noble metals absorb electromagnetic radiation in the visible range of the spectrum (plasmon absorbance), and have been explored for hyperthermia therapy. Gold nanoparticles conjugated with PEG and tumor necrosis factor alpha (TNFα are being developed for targeted cancer therapy (Libutti et al., 2010).

Ferromagnetic materials, such as iron oxide (magnetite, Fe_3_O_4_), iron, cobalt, and nickel offer an additional degree of freedom in the synthesis of nanoparticles for nanomedicine (Arruebo et al., 2007). Very small ferromagnetic nanoparticles (typically < 10 nm) have no intrinsic magnetization in the absence of a magnetic field, and hence do not aggregate in colloidal suspension. These superparamagnetic nanoparticles can be manipulated in an external field providing a simple method for spatial manipulation and washing. Magnetic nanoparticles, such as superparamagnetic iron oxide (SPIO) nanoparticles have been used for magnetic resonance imaging (MRI) and hyperthermia therapy (Yu et al., 2013).

Carbon-based nanoparticles have exploited the small size and unique properties of buckyballs, carbon nanotubes, and grapheme (Yu et al., 2013). Combinations of organic and inorganic materials, taking advantage of specific materials and structures have also been widely explored in multifunctional nanoparticle platforms.

Hybrid nanoparticles with organic and inorganic components or associated combinations of inorganic nanostructures provide further opportunities for introducing multiple functionalities. These systems can exploit the biocompatibility of organic nanoparticles, while still retaining the stability and function of inorganic nanoparticles. Inorganic nanoparticle conjugates allow for multimodal imaging and theranostic applications. Examples include constructs such as liposomes filled with magnetic nanoparticles (Sailor and Park, 2012), coordination polymer nanoparticles (Novio et al., 2013), and metal-organic frameworks (Horcajada et al., 2012).

### Targeting moieties (antibodies, aptamers, small molecules, etc.)

**Figure 1:**
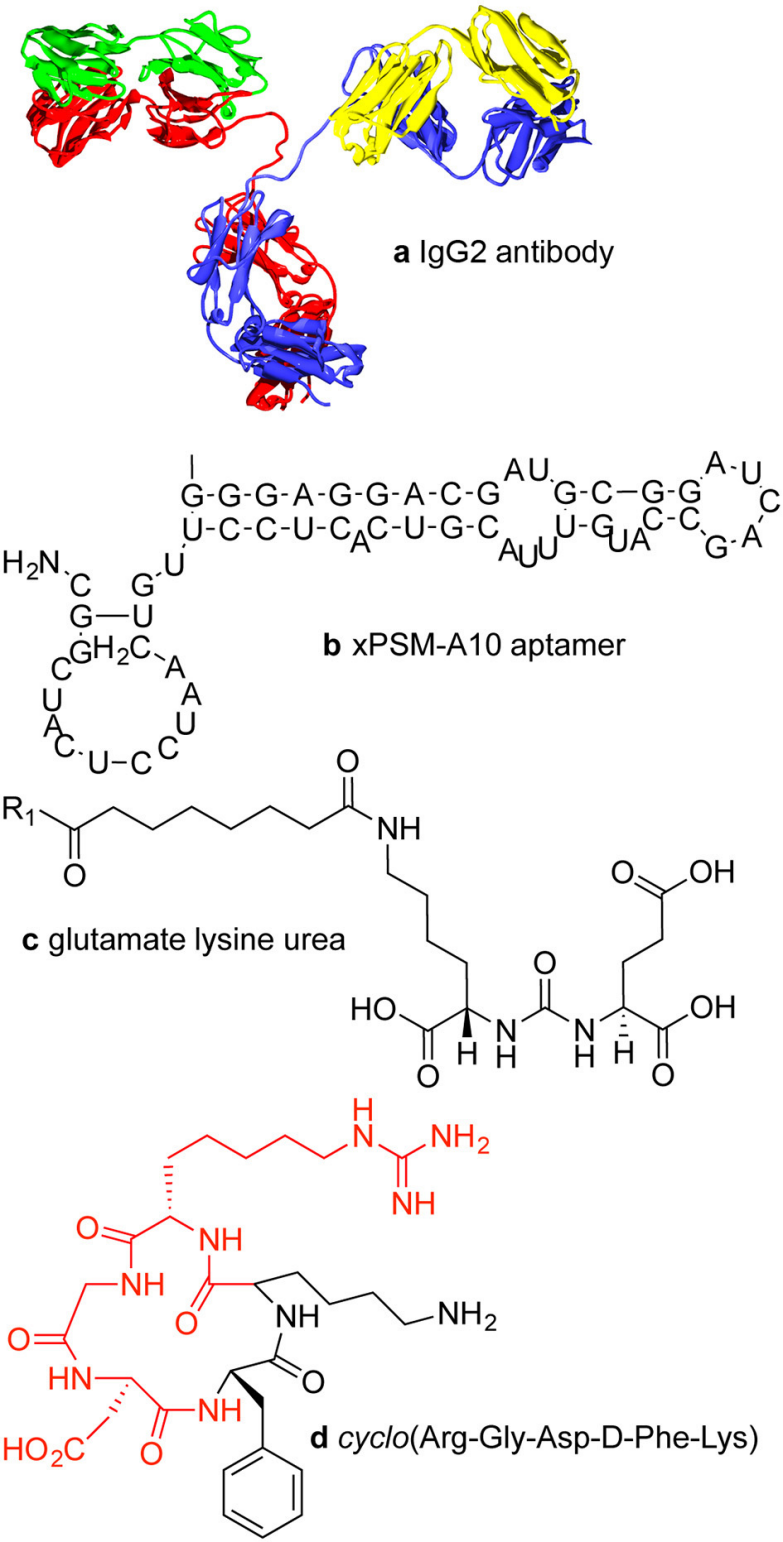
**Examples of targeting molecules. (A)** Antibodies are typically around 150 kDa or about 15 × 5 nm with two antigen binding sites. **(B)** xPSM-A10 is a 18.5 kDa aptamer with a binding affinity of about 10^−8^ M^−1^ for the extracellular portion of the prostate-specific membrane antigen (PSMA) (Lupold et al., 2002). **(C)** The glutamate lysine urea small molecule targets PSMA (473 Da) (Banerjee et al., 2008). **(D)** The RGD peptide sequence (604 Da) binds to cell surface integrins, upregulated in many tumor types.

Active targeting of a nanoparticle is a way to minimize uptake in normal tissue and increase accumulation in a tumor. Strategies for active targeting of tumors usually involve targeting surface membrane proteins that are upregulated in cancer cells (Huynh et al., 2010; Hanahan and Weinberg, 2011). While this strategy is widely used, tumor cell populations are extremely heterogeneous and expression levels can vary significantly. Targeting molecules are typically antibodies (Dill et al., 1994; Arruebo et al., 2009; Chames et al., 2009), antibody fragments (Holliger and Hudson, 2005), aptamers (Keefe et al., 2010; Hu and Zhang, 2013), or small molecules (Figure 1).

Accumulation of a delivery system at a tumor site by the EPR effect is dependent in part on the concentration in the circulation. Processes such as clearance by the MPS or uptake in normal tissue decrease the concentration in circulation and hence decrease the accumulation in the tumor. Active targeting can provide an additional sink for a nanoparticle platform since expression of target molecules is usually differential in that the target is highly expressed in tumor cells but expressed at low levels in other cell types in the vascular system. Since the surface area of the vasculature is much larger than the tumor, active binding in normal tissue can be significant, even for targets that are expressed at relatively low levels (Jain, 2005). Furthermore, targeting moieties may themselves be targets for receptors on phagocytic cells, as described above.

#### Antibodies

Monoclonal IgG antibodies (mAbs) are widely used for protein recognition and targeting since they have two epitope binding sites, high selectivity, and high binding affinity (Chames et al., 2009). Antibodies are the largest of the targeting ligands, approximately 150 kDa or about 15 nm long and about 5 nm in diameter. The binding (dissociation) constants for antibody—antigen interactions vary over a wide range from 10^−6^ to 10^−9^ M, but can be as high as 10^−12^ M for high affinity antibodies (Dill et al., 1994). For targeting applications, the Fc region of the antibody can be a disadvantage if it is accessible to Fc receptors on macrophages, which can lead to increased accumulation in the liver and spleen (Allen, 2002).

#### Antibody fragments

Antigen binding sites represent only a small part of the overall size of antibodies. F(ab′)_2_ fragments retain both antigen binding sites of the antibody coupled by disulfide linkages. Cleavage of the disulfide bond under reducing conditions yields two Fab′ fragments with sulfhydryl groups that can be used for coupling to the targeting platform. Single chain variable fragments maintain only the variable regions (variable light chain and variable heavy chain) of one arm of an antibody.

#### Aptamers

Aptamers are folded single strand oligonucleotides, 25–100 nucleotides in length (8–25 kDa) that bind to molecular targets (Tuerk and Gold, 1990; Keefe et al., 2010). High throughput screening methods can be used for rapid selection of aptamers for specific targets (Bunka and Stockley, 2006). Macugen, approved for use in the treatment of macular degeneration in 2004, is currently the only FDA approved aptamer (Adamis et al., 2006).

#### Small molecules

Small molecules for targeting include peptides, growth factors, carbohydrates, ureas, and receptor ligands (Weissleder et al., 2005). Specific examples include folic acid, transferrin, and the RGD peptide sequence. Folic acid (441 Da) is recognized by the folic acid receptor and is expressed in normal epithelial cells but is overexpressed in many cancer types, especially ovarian, brain, and lung cancers (Kamen and Smith, 2004; Hilgenbrink and Low, 2005; Parker et al., 2005; Chames et al., 2009; Muller and Schibli, 2013; Naumann et al., 2013). Folic acid is essential for amino acid synthesis and hence for cell survival and proliferation, and has a high affinity (*K_d_* < 10^−9^ M) (Hartmann et al., 2007). Transferrin is a chelating protein that regulates the supply of iron into cells via receptor-mediated endocytosis (Kresse et al., 1998). The transferrin receptor is expressed at low levels in most normal tissues but is overexpressed in many tumor types (Daniels et al., 2012). The RGD (Arg-Gly-Asp) peptide is a target for integrins (e.g., α_v_β_3_) on the cell surface (Ruoslahti, 1996; Hynes, 2002). RGD is a component of the extracellular matrix protein fibronectin and promotes cell adhesion and regulates cell migration, growth, and proliferation (Ruoslahti, 1996; Hynes, 2002). A cyclic peptide containing the RGD sequence is widely used for targeting to integrins (Haubner et al., 1996). The upregulation of integrins is promoted by angiogenic factors in several cancer types (Dechantsreiter et al., 1999; Hosotani et al., 2002; Furger et al., 2003; Sheldrake and Patterson, 2009).

## Tumor accumulation and targeting efficiency

In preclinical studies the efficacy of a drug is often determined from the time dependence of tumor size or from the fraction of animals that survive after a candidate therapy. These parameters are particularly useful in assessing the potential therapeutic benefit of a new delivery system but integrate many factors. An additional parameter that is important in assessing the potential efficacy of delivery systems is the tumor accumulation or targeting efficiency—the fraction of an intravenously administered dose that accumulates in a tumor (%ID). Despite the importance of this parameter, very few measurements are reported in the literature.

We have reviewed 40 pre-clinical studies of delivery systems employing passive targeting (Supplementary Table S1), and 34 pre-clinical studies employing active targeting (Supplementary Table S2). Only studies reporting quantitative results of tumor accumulation were selected. Analysis of these pre-clinical studies highlights the need for guidelines to improve the overall impact of research in this field. Despite the importance of pharmacokinetics and tumor accumulation in assessing the efficiency of delivery systems, very few preclinical studies report quantitative results that can be used to develop design rules for nanomedicines.

### Passive targeting

Delivery systems used in pre-clinical studies exploiting passive targeting include liposomes (Harrington et al., 2000; Wang et al., 2006; Soundararajan et al., 2009; Zheng et al., 2009; Huang et al., 2011; Chen et al., 2012a; Coimbra et al., 2012; Hsu et al., 2012; Mahakian et al., 2014) (Kheirolomoom et al., 2010), micelles (Yokoyama et al., 1999; Le Garrec et al., 2002; Kawano et al., 2006; Reddy et al., 2006; Rijcken et al., 2007; Kim et al., 2008; Hoang et al., 2009; Shiraishi et al., 2009; Blanco et al., 2010; Sumitani et al., 2011; Wang and Gartel, 2011; Zhao et al., 2012; Miller et al., 2013; Zhu et al., 2013), gold nanoparticles (Hainfeld et al., 2006; Von Maltzahn et al., 2009; Puvanakrishnan et al., 2012), iron oxide nanoparticles (Ujiie et al., 2011), silica nanoparticles (Chen et al., 2012b; Di Pasqua et al., 2012), carbon-based nanostructures (Liu et al., 2011; Robinson et al., 2012; Rong et al., 2014), quantum dots (Sun et al., 2014), and hybrid nanomaterials (Balogh et al., 2007; Tinkov et al., 2010; Yang et al., 2012) (Paraskar et al., 2012) (Ohno et al., 2013) (Supplementary Table S1).

Of the 40 pre-clinical studies, only a few (4/40) reported tumor accumulation as %ID, while the remainder reported normalized accumulation as %ID/g or %ID/cc. The tumor accumulation varies over a wide range from 0.1 to 35%ID/g at 24 h post-injection. Passive delivery systems are generally pegylated and have sizes in the range from 2 to 200 nm. However, there are no clear trends in terms of identifying physico-chemical parameters that influence the pharmacokinetics or tumor accumulation. Although pegylation is generally assumed to increase circulation time and hence increase tumor accumulation, there is no consistent difference in tumor accumulation between pegylated and non-pegylated delivery systems.

Similarly, there is no obvious dependence on the size or shape of the delivery system. For example, the tumor accumulation of pegylated liposomes around 100 nm in diameter in three studies varied from 0.4 to 11%ID/g (Soundararajan et al., 2009; Kheirolomoom et al., 2010; Hsu et al., 2012). The large variation is likely due to the differences in xenograft cell line, tumor size, and dose. Similarly, tumor accumulation in two pre-clinical studies of 30 nm diameter micelles with different polymer formulations were 1.5%ID/g (Yokoyama et al., 1999) and 9.5%ID/g (Blanco et al., 2010). These two studies used different models (orthotopic vs. xenograft), tumor cell line (A549 vs. C26), tumor size (200 vs. 100 mm^3^), and injected dose (30–50 mg/kg vs. 10 mg/kg). These differences in experimental design limit the ability to compare the two different micelle formulations. These examples highlight the difficulty in comparing pre-clinical trials due to the variability in experimental design.

### Active targeting

Targeted delivery systems used in quantitative pre-clinical studies include silica (Benezra et al., 2011; Tang et al., 2012; Chen et al., 2013), gold (Melancon et al., 2008; Lu et al., 2009, 2010; Chanda et al., 2010; Choi et al., 2010; Morales-Avila et al., 2011; Chattopadhyay et al., 2012), liposomes (Iyer et al., 2011; Helbok et al., 2012; Petersen et al., 2012), micelles (Hu et al., 2008; Penate Medina et al., 2011; Zhang et al., 2011b; Fonge et al., 2012; Helbok et al., 2012) (Rossin et al., 2005; Khemtong et al., 2009; Zhan et al., 2010; Poon et al., 2011; Zhang et al., 2011a; Xiao et al., 2012), iron oxide (Natarajan et al., 2008; Kumar et al., 2010; Yang et al., 2011), graphene (Hong et al., 2012; Cornelissen et al., 2013; Shi et al., 2013), gadolinium (Oyewumi et al., 2004), polymer nanocarriers (Kunjachan et al., 2014), nanoemulsions (Ohguchi et al., 2008), quantum dots (Gao et al., 2010), and hybrid (Cheng et al., 2014) (Supplementary Table S2). Similar to passive targeting, few studies (3/34) report %ID rather than %ID/g. The most common targeting ligands are antibody based (9/34 studies), the RGD peptide sequence (10/34), and folate (5/34). Targeting efficiencies obtained using RGD peptides, folate, antibodies, and antibody fragments are typically between 1 and 15%ID/g (Supplementary Table S2).

Assessing the efficiency of a targeting ligand in increasing tumor accumulation is complicated by the different control experiments used in these studies. The contribution of passive targeting was assessed by measuring tumor accumulation of the delivery system without attachment of the targeting ligand (20/34), with attachment of a non-specific ligand (2/34), pre-injection with a blocking molecule or treatment (10/34), or with a xenograft formed from a cell line that did not express the target molecule (2/34). Several studies (4/34) did not report a control experiment. Each control experiment has advantages and disadvantages. For example, removing a targeting ligand from a delivery system may alter the physico-chemical properties and hence change the pharmacokinetics. As described in more detail below, xenografts formed from different cell lines may have significantly different vascularization and hence the rate of extravasation to the tumor site by the EPR effect may be significantly different. Pre-injection with a blocking molecule may not completely prevent binding to the target molecule or may reduce binding in normal tissue. To account for these potential complications, a few studies (3/34) used multiple controls.

Of the 30 pre-clinical studies that reported control experiments, 33% (10/30) showed less than a two-fold increase in targeting compared to the control, and 50% (15/30) showed an increase in tumor accumulation of more than 2%ID/g with the targeting ligand. For example, a tumor accumulation of 9%ID was reported for SPIONS with anti-ChL6 2 days post-injection compared to 1% without the targeting antibody (Natarajan et al., 2008). A tumor accumulation of 7 ± 1%ID was reported for gadolinium nanoparticles with a folate targeting ligand, and 9 ± 4%ID in the control with no targeting ligand (Oyewumi et al., 2004). While active targeting of a delivery system to a tumor site has the potential to reduce unwanted side effects, these studies highlight the difficulties in assessing targeting efficiency due to the large differences in experimental design and the range of controls used to assess the contribution of passive targeting.

### Tumor accumulation

In general, the uptake of a delivery system in a tumor tends to increase post-injection but then decreases at longer times (Supplementary Table S1 and S2). For example, tumor accumulation of radiolabeled liposomes increased to 11.3%ID/g over the first 24 h, then decreased to 6.1%ID/g after 72 h (Hsu et al., 2012). Tumor accumulation of self-activating quantum dots increased to 13%ID/g over the first 24 h, but decreased to 11%ID/g after 42 h (Sun et al., 2014). Similarly, tumor accumulation of pegylated micelles with a gelatinase binding peptide was reported to increase to almost 18% ID/g over the first 6 h, but decreased to 2% ID/g after 24 h (Penate Medina et al., 2011). Tumor accumulation of gold nanoparticles with the RGD peptide increased to 3.65% ID/g over the first hour followed by a decrease by almost half to 1.94% ID/g 24 h post-injection (Morales-Avila et al., 2011). The details of the time dependence of tumor accumulation are important in understanding the pharmacokinetics, the EPR effect, and the limitations to accumulating a drug at the tumor site. In many studies, an insufficient number of time points precludes detailed analysis of pharmacokinetics and tumor accumulation.

The cell line used in forming a xenograft can have significant influence on tumor accumulation and efficacy. In the 74 quantitative pre-clinical trials reviewed here, 35 different cell types were used to form xenografts. The most common cell lines were the 4T1 murine breast cancer cell line (10/71) and the C26 colon carcinoma cell line (10/71), both of which form highly vascularized tumors. Tumor accumulation of micelles with the RGD peptide was 6%ID/g in a mouse model with a C26 xenograft and 3%ID/g with a less leaky BxPC3 xenograft (Kunjachan et al., 2014), highlighting the need for standardization of cell lines.

Tumor size can have a significant influence on tumor accumulation. For example, a study using radiolabeled liposomes compared targeting efficiency among tumors of different sizes using the KB cell line (Harrington et al., 2000). The tumor accumulation for small tumors (≤0.1 g) was around 15%ID/g, whereas for larger tumors (≥1 g) was only 3%ID/g.

## Guidelines for pre-clinical studies of delivery systems

While the physico-chemical properties of delivery systems are expected to exert a significant influence on pharmacokinetics, tumor accumulation, and biodistribution, there are numerous problems in comparing pre-clinical studies. In particular, differences in cell line and tumor size, dose, lack of good pharmacokinetics data, and differences in reporting make meta-analysis extremely difficult and are a limitation to progress in the field (Table 4). Similarly, physico-chemical properties of the delivery system such as size, surface properties (i.e., pegylation), zeta potential, targeting ligand density, and stability in blood or serum at physiological temperature are not uniformly reported.

**Table 4:** **Summary of limitations to pre-clinical studies of nanomedicines that hinder broad assessment of design rules**.

**Problem**	**Solution**
Total tumor accumulation (%ID) is not always reported	Report tumor accumulation as %ID (and %ID/g)
Inconsistent reporting of tumor size/weight	Report tumor size/weight
Inconsistent reporting of dose	Report dose as total number of nanoparticles injected Along with other parameters such as drug loading, drug concentration (and/or drug amount), and activity of dose (gamma counter)
Inconsistent reporting of physico-chemical properties	Report standard physico-chemical properties (e.g., size, zeta potential, surface coating, stability under physiological conditions)
Tumor accumulation reported at different time points	Report tumor accumulation at standard time points (e.g., 1 and 24 h post-injection). Detailed pharmacokinetics (concentration in blood and tumor) at multiple time points is preferred
Variation in tumor characteristics (type, size, vascularization, etc.)	Standardize tumor type and size (e.g., C26 or 4T1; 1 cm diameter) More difficult for active targeting depending on target molecule
Variation in controls used in active targeting	Report control studies for delivery system with no targeting ligand and any differences in physico-chemical properties. Report other control studies as necessary
Variation in animal models (mouse, rat, etc.) and differences in drug concentration compared to humans	Use mouse xenograft model for initial pre-clinical studies
Different detection methods used to assess tumor accumulation	Perform validation using other method(s)

For example, results are usually reported as percent of initial administered dose per gram of tumor (%ID/g), which is only useful if the tumor mass is also reported. For example, a tumor accumulation of 10%ID/g is 10% of the initial dose for a 1 g tumor but 1% of the initial dose for a 0.1 g tumor. These differences are significant in terms of the efficiency of delivery and minimizing unwanted side effects in normal tissue. In some cases tumor characteristics such as tumor diameter or approximate tumor volume are reported, however, these parameters can only be used to estimate the absolute percentage of the initial dose.

Mouse models are widely used for research studies of disease progression and the development of new therapies (Frese and Tuveson, 2007). Rat and rabbit models are also commonly used for pre-clinical studies. Standard tumor models include subcutaneous xenografts of human cell lines or explants, orthotopic xenografts, and genetically engineered mouse models (Frese and Tuveson, 2007; Chen et al., 2012a). While these models are invaluable for pre-clinical studies, differences in physiology can lead to differences in circulation and tumor accumulation compared to humans (Steichen et al., 2012).

Xenografts represent a relatively straightforward model to study the pharmacokinetics, tumor accumulation, and biodistribution of a nanomedicine, however, tumor characteristics vary considerably with cell line and size (Harrington et al., 2000; Jain and Stylianopoulos, 2010). The density and vascularization of tumors of similar size can also vary significantly. Highly invasive cell lines often form more highly vascularized tumors, for example xenografts of colon cancer cell lines have vasculature that is much more leaky than pancreatic cancer cell lines. Therefore, tumor uptake by the EPR effect is expected to be strongly dependent on the cell line used. Establishing a standard cell line and tumor size for xenografts would greatly enhance comparison of pre-clinical trials of delivery systems (Table 4). While this is feasible for passive delivery systems, active targeting often requires the use of specific cell lines that overexpress a particular biomarker.

Tumor accumulation is usually measured using a gamma counter, positron emission tomography (PET), or inductively coupled plasma mass spectroscopy (ICP-MS). The methods using a gamma counter or PET require that a suitable radiolabel is conjugated to the drug delivery platform. With a gamma counter, the radioactivity of the resected tumor is measured and compared to the radioactivity of the dose. To determine the tumor accumulation from PET scans, reconstructed 3D regions of interest are drawn around the tumor. The activity per unit mass can then be determined after correcting for decay and tissue density. An alternative to using a radiolabel to measure the tumor accumulation is to use ICP-MS to determine the amount of one or more elemental components in the delivery system and to compare to the initial dose. However, this method requires a component of the delivery system to be distinguishable from biological matter. In most pre-clinical studies only one of the methods is used to determine pharmacokinetics and tumor accumulation and hence there is no independent verification.

Tumor accumulation is expected to be dependent on the dose and time post-injection, and hence time-course studies at different doses are important for full characterization. In many cases, tumor accumulation is determined only at one or two time points therefore limiting analysis of the pharmacokinetics which is crucial for developing design rules.

## Summary

Nanoparticle-based delivery systems provide new opportunities to overcome the limitations associated with traditional drug therapy and to achieve both therapeutic and diagnostic functions in the same platform. The efficiency of drug or gene delivery to a tumor site is dependent on the physico-chemical properties of the delivery platform and a range of physiologically imposed design constraints including clearance by the mononuclear phagocyte system and extravasation from circulation at the tumor site by the enhanced permeability and retention effect.

The lack of uniformity in pre-clinical trials of nanoparticle-based delivery systems has prevented systematic comparison of these studies and has been an impediment to developing design rules for new systems or specific applications. Of the large number of pre-clinical trials, surprisingly few report quantitative data on parameters that would be useful in developing design rules for nanomedicines. The poor experimental design and variability of experimental conditions also contribute to slow development of the field and the lack of clinical impact. We highlight some of the problems with pre-clinical trials nanoparticle-based delivery systems and suggest some solutions to increase the impact of individual studies.

### Conflict of interest statement

The authors declare that the research was conducted in the absence of any commercial or financial relationships that could be construed as a potential conflict of interest.
