# Supplementary material for: Nanomedicines for cancer therapy: state-of-the-art and limitations to pre-clinical studies that hinder future developments
Source: Front Chem. 2014 Aug 25;2:69. doi: 10.3389/fchem.2014.00069 (PMC4142601; doi:10.3389/fchem.2014.00069)
Supplement: Supplementary file 1 [file DataSheet1.PDF]

Supplementary Table S1 – Quantitative studies of passively targeted nanoparticles

Nanoparticle Type	Size	Cargo	Composition	Tumor type	Tumor size/ weight	Dose	Targeting Efficiency	Method	Reference
**Block copolymer micelles**	30 nm	Adriamycin	PEG-P(Asp)	Colon 26 xenografts in mice	100 mm^3	10 mg/kg	4 ± 0.4 %ID/g @ 1 hr, 5 ± 1 %ID/g @ 4 hr, 9.5 ± 1 %ID/g @ 24 hr, 9 ± 0.5 %ID/g @ 48 hr	LSC-1000	(Yokoyama et al., 1999)
**Block copolymer micelles**	58 nm	^{111}In	PEG-e-caprolactone-p-SCN-Bn-DTPA-^{111}In	MDA-MB-231 xenografts in female nude mice	150 mm^3	250 mg/kg	9 ± 2 %ID/g @ 48 hr	MicroSPECT, CT, gamma counter	(Hoang et al., 2009)
**Carbon nanotubes**	41.6 nm	SWNT	SWNT+C_{18}-PMH-mPEG	4T1 xenografts in Balb/c mice	20-50 mm^3	200µL of 0.35 mg/mL	30 %ID/g @ 100 hr	Raman scattering	(Robinson et al., 2012)
**Composite nanodevices**	5 nm	Gold	PAA dendrimers + gold nanoparticles, positively charged surface	B16F10 xenografts in male mice, MatLyLu xenografts in mice	500 mm^3	16 mg/kg	2 ± 0.1 %ID/g @ 5 min, 6 ± 0.1 %ID/g @ 1 hr, 6.5 ± 1.5 %ID/g @ 24 hr, 3 ± 0.2 %ID/g @ 96 hr	Neutron irradiation	(Balogh et al., 2007)
**Coordination Polymer**	100-200 nm	Oxaliplatin, FITC	PIMA-GA-DACH-platinum	4T1 xenografts in mice	50 mm^3	5mg/kg and 15 mg/kg	0.02 %ID/g for 5 mg/kg dose, 0.05 %ID/g for 15 mg/kg dose	ICP-MS	{Paraskar, 2012 #188}
**Core-crosslinked micelles**	45.8 nm	Boron	acetal-PEG-b-PLA-MA + polymerizable carborane crosslinker	Colon 26 xenografts in mice	100 mm^3	0.2 mg boron atoms/kg	Crosslinked: 5.4 %ID/g, noncrosslinked: 1.4 %ID/g	ICP-MS	(Sumitani et al., 2011)
**Core-crosslinked polymeric micelles**	24 ± 8 nm	D-glucosamine, NIRF, ^{111}In	Proprietary	A431 xenografts in mice	8-12 mm in diameter	10 µCi/mouse, 1.3 x 10^{12} particles/mouse	2.75 ± 1 %ID/g @ 4 hr, 2.62 ± 0.8 %ID/g @ 24 hr	Gamma counter	(Zhu et al., 2013)
**Core-crosslinked polymeric micelles**	24 ± 8.9 nm	Triethoxysilane, NIRFSi, ^{111}In	Block copolymer PEG + triethoxysilane hydrophobic block	CT-26 xenografts in female nude mice	8-10 mm in diameter	1 mg/mouse, 195 µCi	8.5 ± 1 %ID/g @ 24 hr, 8.5 ± 2 %ID/g @ 48 hr (SE)	SPECT, NIRF	(Zhao et al., 2012)
**Core-crosslinked polymeric micelles**	68 ± 7 nm	^{3}H-acetate	mPEG5k + mPEG-b-p(HEMAm-Lac_n)	14C xenografts in mice		200 µL of 10 mg/mL micelles	Crosslinked: 5 %ID/g @ 1 hr, 6 %ID/g @ 4 hr, 7 %ID/g @24 hrs, 6 %ID/g @ 48 hr. Non-crosslinked: 3 %ID/g @ 1 hr, 2.5 %ID/g @ 4 hr, 1 %ID/g @ 24 hr, 0 %ID/g @ 48 hr	Gamma counter	(Rijcken et al., 2007)
**Core-crosslinked polymeric micelles and long-circulating liposomes**	70-100 nm	Dexamethasone	DPPC + chol + DSPE-PEG2k	B16F10 xenografts in male mice	100 mm^3	1 x 10^6 cpm	10 %ID/g for micelles, 5 %ID/g for LC-liposomes @ 24 hr and 48 hr	Liquid scintillation counter	(Coimbra et al., 2012)
**Core-shell nanoparticles**	50 nm	Polypyrrole	Core-shell silica and polypyrrole	4T1 xenografts in Balb/c mice	60-70 mm^3	10 mg/kg	5 %ID/g @ 1 hr, 5 %ID/g @ 6 hr, 4 %ID/g @ 24 hr	ICP-AES	(Chen et al., 2012b)
**Graphene**	50 nm	Iron oxide nanoparticles, ^{125}I	RGO-IONP-PEG	4T1 xenografts in mice	50 mm^3	4 mg/kg	5 %ID/g @ 48 hr	PET	(Yang et al., 2012)
**Graphene**	50 nm	HPPH, ^{64}Cu	Graphene-PEG-HPPH	4T1 xenografts in nude mice	100 mm^3	3.7 MBq	3 %ID/g @ 24 hr	PET	(Rong et al., 2014)

**Hybrid nanoparticles**	100 ± 11 nm and 210 ± 23 nm	^{125}I	Fe_3O_4 core, poly(PEGMA) brushes	Colon26 xenografts in mice	7 mm in diameter		21 % ID/g for high MW dendrimer, 3 % ID/g for low MW dendrimer @ 24 h	Gamma counter	{Ohno, 2013 #189}
**Liposomes**		^{111}In, DTPA	Pegylated, proprietary	KB xenografts in mice	varied	10-20 µCi	mean: 7.2 ± 6.6 %ID/g @ 24 hr, tumor size dependence: ≤0.1 g, 15.1 ± 10.8 %ID/g, 0.1-1 g, 5.9 ± 2.2 %ID/g, ≥1 g, 3 ± 1.3 %ID/g	Gamma counter	(Harrington et al., 2000)
**Liposomes**	80 nm	^{188}Re, BMEDA	DSPC + chol + DSPE-PEG2k	orthotopic Fischer344/F98 glioma tumor-bearing rats	50-100 mm^3	14.8 MBq	0.28 ± 0.09 %ID/g @ 1 hr, 0.75 ± 0.08 %ID/g @ 4 hr, 1.95 ± 0.35 %ID/g @ 24 hr, 1.37 ± 0.39 %ID/g @ 48 hr, 1.09 ± 0.08 %ID/g @ 72 hr (SD)	Gamma counter	(Huang et al., 2011)
**Liposomes**	80 nm	Iohexol and gadoteridol	DPPC + chol + DSPE-PEG2k	VX2 xenografts in NZ white rabbits	5000-10000 mm^3	1785 mg/kg iohexol, 40 mg/kg gadoteridol	0.7 ± 0.1 %ID @ 24 hr, 0.9 ± 0.3 %ID @ 48 hr, 0.9 ± 0.3 %ID @ 72 hr (SD)	CT quantitative imaging	(Zheng et al., 2009)
**Liposomes**	83 nm	BMEDA, ^{188}Re, 5-FU	Pegylated, proprietary	CT26-luc xenografts in mice	100 mm^3	2.96 MBq, 100 µL liposomes, 0.44 µmol phospholipid	4.98 ± 0.57 %ID/g @ 1 hr, 5.67 ± 0.46 &ID/g @ 4 hr, 5.40 ± 0.42 %ID/g @ 16 hr, 5.46 ± 0.37 %ID/g @ 24 hr, 3.53 ± 0.26 %ID/g @ 48 hr, 1.98 ± 0.19 %ID/g @ 72 hr (SEM)	Gamma counter	(Chen et al., 2012a)
**Liposomes**	83 nm	^{188}Re, BMEDA	Pegylated, proprietary	LS-174T xenografts in mice	50-70 mm^3	2.59 MBq, 100 µL liposomes, 0.7 µmol phospholipid	2.61 ± 0.6 %ID/g @ 1 hr, 4.26 ± 0.7 %ID/g @ 4 hr, 11.27 ± 0.99 %ID/g @ 24 hr, 7.05 ± 0.56 %ID/g @ 48 hr, 6.07 ± 0.35 %ID/g @ 72 hr (SE)	Gamma counter	(Hsu et al., 2012)
**Liposomes**	90 nm	Doxorubicin, ^{186}Re	Doxil	HNSCC xenografts in nude rats	1 g	555 MBq/kg	0.36 ± 0.33 %ID/tumor @ 120 hrs (SD)	Gamma counter, microSPECT, CT	(Soundararajan et al., 2009)
**Liposomes**	100 nm	Copper-doxorubicin complex	Doxil	Bilateral Met1 xenografts in mice	≥100 mm^3	6 mg doxorubicin/kg	10 %ID/g	ICP-MS	(Kheirolomoom et al., 2010)
**Liposomes**	100 nm	^{64}Cu	HSPC + chol + DSPE-PEG2k-OMe/6-BAT-PEG-lipid	DMBA-induced tumors in hamsters	2 mm in diameter		3.5 ± 1.4 %ID/cc @ 24 hr	PET	(Mahakian et al., 2014)
**Liposomes**	110 nm	^{177}Lu	DTPA/PEG	Colon 26 xenografts in mice	562 ± 142 mm^3, 20 g	3.7 MBq	0.61 ± 0.32 %ID/g @ 1 hr, 1.53 ± 0.6 %ID/g @ 4 hr, 3.97 ± 1.75 %ID/g @ 24 hr, 4.68 ± 1.55 %ID/g @ 48 hr, 3.23 ± 1.7 %ID/g @ 72 hr (SD)	Gamma counter	(Wang et al., 2006)
**Mesoporous silica nanoparticles**	400 nm	^{165}Ho	MCM-41 + ^{165}Ho	A549-luciferase orthotopic injection in mice		150 µCi	4.5 ± 3.9 %ID/g @ 24 hr, 58.8 ± 34.7 %ID/g @ 1 week	Gamma counter	(Di Pasqua et al., 2012)
**Micelles**	30 nm	β-lapachone	PEG-PLA	A549 orthotopic lung cancer in mice	200 mm^3	30-50 mg/kg	1.8 ± 0.2 %ID/g @ 2 hr, 1.9 ± 0.2 %ID/g @ 24 hr (SE)	IC liquid scintillation counter	(Blanco et al., 2010)
**Micelles**	43 ± 24 nm	Etoposide, ^{99m}Tc	Polysorbate 20	Dalton's lymphoma xenografts in Balb/c mice	1 cm^3	100 µCi	10.5 ± 2 %ID @ 1 hr, 5.5 ± 2 %ID @ 6 hr, 5 ± 1 %ID @ 24 hr (SD)	Gamma ray spectrometry	(Reddy et al., 2006)
**Micelles**	100 nm	Thiostrepton	Amphiphilic lipid-PEG	MDA-MB-231 and HepG2 xenografts in mice	0.25 g	1.8 mg thiostrepton/ animal	16 ± 4 %ID @ 4 hr, 35 ± 2 %ID @ 24 hr (SEM)	LC/MS	(Wang and Gartel, 2011)

**Micelles**	110 nm	IFF, ^{111}In	PEG-PDLLA	AR42J xenografts in mice	1 cm^3	100 µL of micelle solution	1 %ID/g up to 120 mins	Gamma counter	(Miller et al., 2013)
**Micelles**	192 ± 13 nm	Camptothecin	PEG-poly(benzyl aspartate)	C26 xenografts in mice	100 mm^3	2.5 mg/kg	1.3 %ID/g @ 24 hr	HPLC	(Kawano et al., 2006)
**Nanoparticles**	1.9 nm	Gold	Gold, proprietary	EMT-6 xenografts in mice	5 mm in diameter	0.01 mL/g of 270 mg Au/cm^3	4.2 ± 0.25 %ID/g @ 20 mins, 2.5 ± 0.1 %ID/g @ 1 hr, 3 ± 0.1 %ID/g @ 5.5 hr, 3.2 ± 0.5 %ID/g @ 11 hr, 2.7 ± 0.1 %ID/g @ 24 hr	Graphite furnace atomic absorption	(Hainfeld et al., 2006)
**Nanoparticles**	50 nm	Iron oxide	Iron oxide + PEG-poly(4-vinylbenzylphosphonate)	C-26 xenografts in mice	>100 mm^3	84 mg Fe/kg	1 %ID/g @ 5 mins, 5 %ID/g @ 1 hr, 17 %ID/g @ 4 hr, 17 %ID/g @ 24 hr, 15 %ID/g @ 48 hr, 14 %ID/g @ 96 hr	ICP-MS	(Ujiie et al., 2011)
**Nanorods**	13x47 nm	Gold	Gold, PEG	MDA-MB-435 xenografts in mice		20 mg Au/kg	7 %ID/g @ 72 hr	ICP-MS	(von Maltzahn et al., 2009)
**Nanorods**	24 x 7 nm	Gold	Gold+mPEG-SH	A431 xenografts in mice	8-10 mm in diameter	2 x 10^{11} particles/µL	1.35 ± 0.29 %ID/g @ 24 hr	Neutron activation analysis	(Puvanakrishnan et al., 2012)
**Nanoshells**	120 nm	Gold	Colloidal silica + gold + SH-PEG	A431 xenografts in mice	8-10 mm in diameter	2.74 x 10^8 particles/µL	0.118 ± 0.027 %ID/g @ 24 hr	Neutron activation analysis	(Puvanakrishnan et al., 2012)
**Phospholipid microbubbles**	1 µm	Doxorubicin	DPPC + DPPG + DPPE-PEG2k	DSL6A xenografts in Lewis rats	5-8 mm in diameter	140 µg doxorubicin	0.9 ± 1 %ID/g	HPLC	(Tinkov et al., 2010)
**Polyelectrolyte complex micelles**	50-80 nm	siRNA	VEGF siRNA-PEG/PEI	PC3 xenografts in female nude mice	50 mm^3	3 nmol siRNA	0.3 ± 0.01 %ID/g @ 4 hr, 0.5 ± 0.1 %ID/g @ 24 hr (SD)	PCR, fluorescence measurements	(Kim et al., 2008)
**Polymeric micelles**	30 nm	Aluminum chloride phthalocyanine	NIPAM + N-vinyl-2-pyrrolidone	EMT-6 xenografts in male mice	25 mm^3	2 µmol/kg of drug	1 %ID/g @ 1 hr, 1 %ID/g @ 24 hr, 2 %ID/g @ 48 hr	Fluorescence spectroscopy	(Le Garrec et al., 2002)
**Polymeric micelles**	42 nm	Gadolinium	PEG-b-poly(L-lysine)	Colon 26 xenografts in mice	50-100 mm^3	0.05 mmol Gd/kg	6.1 ± 0.3 %ID/g @ 24 hr	ICP	(Shiraishi et al., 2009)
**Quantum dots**	14-28 nm	^{64}Cu	CdSe/ZnS + PEG	U87MG xenografts in mice	100 mm^3	25 µg QDs	5 ± 1 %ID/g @ 1 hr, 12.5 ± 5 %ID/g @ 17 hr, 13.5 ± 4 %ID/g @ 24 hr, 11 ± 3 %ID/g @ 42 hr	PET, ICP-MS	(Sun et al., 2014)
**SWNTs**	200 nm	$PMHC_{18}$	SWNT-PEG-$PMHC_{18}$	4T1 xenografts in Balb/c mice	50-100 mm^3	200 µL of 0.5 mg/mL solution	17 %ID/g for 100%-2kPEG-$PMHC_{18}$ @ 48 hr	Raman spectroscopy	(Liu et al., 2011)

Supplementary Table S2 - Quantitative studies of actively targeted nanoparticles

NP Type	Size	Cargo	Targeting	Composition	Dose	Tumor type	Tumor size/weight	Targeting Efficiency	Method	Reference
**Graphene**	27.0 ± 0.9 nm with Ab	^{64}Cu	aCD105 antibody	PEG	5-10 MBq (= 135-270 µCi)	4T1 xenografts in mice	5-8 mm in diameter	4.5 ± 0.6 %ID/g with Ab, 2.7 ± 0.3 %ID/g blocked, ~2.5% @ 24h (SD)	PET, gamma counter	(Hong et al., 2012)
**Graphene**	64 nm	^{111}In	aHER2 (trastuzumab)		10 µg (5 MBq, 135 µCi)	231/H2N xenografts in mice	500 µL	15.0 ± 3.7 %ID/g without blocking, 1.45 ± 0.04 %ID/g with blocking, 0.11 ± 0.04 %ID/g NP-IgG @72h (SD)	Gamma counter	(Cornelissen et al., 2013)
**Graphene**	22.3 ± 3.2 nm	^{64}Cu	aCD105 antibody	PEG	5-10 MBq	4T1 xenografts in mice	6-8 mm in diameter	4 .5 ± 0.4 %ID/g RGO-TRC105, 2.5 ± 0.2 %ID/g RGO-TRC105 blocked, 1.6 %ID/g RGO @24h	Gamma counter	(Shi et al., 2013)
**Hollow nanoshells**	34.4 nm without Ab; 37.0 nm with Ab	^{111}In	aEGFR C225 Antibody	Gold	10 µCi/mouse ($7.3x10^{10}$ particles/mL in 0.13 mL)	A431 xenografts in mice	4-6 mm in diameter	6.81 ± 2.64 %ID/g C225-HauNS, 4.60 ± 1.31 %ID/g CTRL IgG-HAuNS @ 24h (SD)	Gamma counter	(Melancon et al., 2008)
**Hollow nanospheres**	43.5 ± 2.3 nm	^{111}In	α-melanocyte-stimulating hormone analog	Gold, pegylated	$2x10^{12}$ particles/mouse (40 µCi/mouse in 0.2mL)	B16/F10 xenografts in mice	4-6 mm in diameter	12.6 ± 3.1 %ID/g targeted, 4.3 ± 1.2 %ID/g nontargeted @ 4 hr (SD)	Gamma counter, microPET	(Lu et al., 2009)
**Hollow nanospheres**	45 nm	si-RNA, ^{64}Cu	Folate	Gold, PEG	0.25 µmol SiRNA/kg	HeLa xenografts in mice	<1.2 cm	5.26 ± 1.25 %ID/g F-NP, 1.11 ± 0.5 %ID/g NP CTRL @ 6 hr (SD)	Gamma counter	(Lu et al., 2010)
**Liposomes**	~120 nm	^{64}Cu	Somastatin peptide analog TATE TATE-mal to thiol-PEGlipid	DSPE, DSPE-PEG, chol	10 mg lipid/kg	NCI-H727 xenografts in female mice	< 0.5 g	1.7 ± 0.1 %ID/g @ 1h TATE, 2.7 ± 0.2 %ID/g @ 8h TATE 5.1 ± 0.3 %ID/g @ 24h TATE, 5.0 ± 0.4 %ID/g @ 48h TATE, 1.9 ± 0.1 %ID/g @ 1h CTRL, 3.0 ± 0.2 %ID/g @ 8h CTRL 5.9 ± 0.2 %ID/g @ 24h CTRL, 6.2 ± 0.2 %ID/g @ 48h CTRL (SE)	Gamma counter, PET	(Petersen et al., 2012)
**Liposomes**	110-120 nm	^{111}In	scFv	Pegylated, POPC, DMPE-DTPA, DSPE-mPEG2000, DSPE-mPEG2000-mal	18.5-37 MBq (0.5-1 mCi)	M28 epithelial and VAMT1 sarcomoid xenografts in mice	3-5 mm in diameter	3.24 ± 0.24 %ID/g @ 24h M28, 4.01 ± 0.39 %ID/g @ 48h M28, 0.97 ± 0.48 %ID/g @ 48h M28 CTRL, 3.86 ± 0.23 %ID/g @ 24h VAMT1, 4.69 ± 0.72 %ID/g @ 48h VAMT1, 0.4 ± 0.42 %ID/g @ 48h VAMT CTRL (SD)	Gamma counter	(Iyer et al., 2011)
**Liposomes and Micelles**	97 ±2 nm; 20 ± 24 nm	^{111}In	Somatostatin analogue tyrosine-3-octreotide	POPC, Lyso-PG, DSPE-PEG200, DMPE-DTPA	12.5 µg liposome/kg rat; <3 µg micelle/kg rat	AR42J xenografts in rats	20-120 mm^3	~2.5 %ID/g @ 1h, 4h, liposome and micelle; ~0.75 %ID/g all blocked	Gamma camera	(Helbok et al., 2012)

**Mesoporous Silica**	80 nm	Doxorubicin and ^{64}Cu	aCD105 antibody	PEG	5-10 MBq	4T1 xenografts in mice	5-8 mm in diameter	5.4 ± 0.4 %ID/g silica-TRC105, 2.6 ± 0.2 %ID/g silica @5h (SD)	Gamma counter	(Chen et al., 2013)
**Micelles**	60 nm and 15 nm	^{111}In	hEGF Fab	PEG-PCL block copolymer	250 mg/kg (5-7 MBq/mouse)	MDA-MB-468 xenografts in female nude mice	5-10 mm in diameter	2.0 %ID/g 15 nm no hEGF, 0.5 %ID/g 15 nm with hEGF, 4.1 %ID/g 60 nm no hEGF, 0.7 %ID/g, 60 nm, with hEGF	Gamma counter	(Fonge et al., 2012)
**Micelles**	128 ±10 nm	Paclitaxel, ^{125}I	RGD peptide	PLA-PEG	15 mg/kg	MDA-MB-435 xenografts in mice	Tumor weight=a^{2}b/2 in mg<br>300 mm^3	3.53 ± 0.14 %ID/g PLA-PEG-RGD, 0.84 ± 0.09 %ID/g PLA-PEG (SD)	Gamma counter	(Hu et al., 2008)
**Micelles**	14 nm	Doxorubicin, ^{125}I	Gelatinase binding peptides	DSPE, CTT2-PEG3400-DSPE	200 µg/mouse	OV-90 xenografts in male mice	65-200 mm^2	~14 %ID/g @ 1.5h, 17.6 %ID/g @ 6h, ~2 %ID/g @24h	Gamma counter	(Penate Medina et al., 2011)
**Micelles**	23 nm	^{111}In, Cy7dye-like molecules	EphB4-binding peptide	Core-crosslinked, polymeric, proprietary	1x10^{14} micelles/mouse	PC-3M prostate (EHB4+) and A549 lung (EHB4-) xenografts in mice	5-6 mm in diameter	2.87 %ID/g PC3-M, 1.42 %ID/g PC-3M blocked, 1.37 %ID/g A549 @ 24h	Gamma counter,	(Zhang et al., 2011b)
**Micelles**	25 nm	^{111}In	Annexin A5 protein	Core-crosslinked polymeric	5x10^{13} NP/ mouse	EL4 lymphoma and MDA-MB-468 breast xenograft, pretreated with cyclophosphamide and etoposide for apoptosis, in mice	5-6 mm in diameter	8.01 %ID/g pretreated for apoptosis, 3.2 %ID/g untreated @ 48h	Gamma counter	(Zhang et al., 2011a)
**Micelles**	35 nm	^{125}I	Cyclic RGD	PEG-PLA	35 µCi	U87MG xenografts in mice	0.4-0.6 cm in diameter	1.4 ± 0.086 %ID/g not blocked, 0.44 ± 0.087 %ID/g blocked @ 24 hr (SD)	Gamma counter	(Zhan et al., 2010)
**Micelles**	80 ± 6 nm	Paclitaxel, ^{111}In	Folate	LDP micelles, pegylated	10 mg/kg mouse	KB xenografts in mice	w^2*l/2<br>500 mm^3	5 ± 0.4 %ID/g @ 5 min NP-folate, 10 ± 2 %ID/g @ 3 day NP-folate, 5 ± 0.5 %ID/g @ 5 min NP-CTRL, 1 ± 0.3 %ID/g @ 3 day NP-CTRL (SD)	Scintillation counter	(Poon et al., 2011)
**Micelles**	65 nm	Doxorubicin and ^{64}Cu	cRGD peptide	Block copolymer, H40-P(LG-Hyd-DOX)-6-PEG	5-10 MBq	U87MG xenografts in mice	6-8 mm in diameter	4.1 ± 0.5 %ID/g @ 30 min NP-cRGD, 5.7 ± 1.2 %ID/g @ 4 h NP-cRGD, 3.7 ± 0.8 %ID/g @ 16 h NP-cRGD, 3.1 ± 0.2 %ID/g @ 24 h NP-cRGD, 2.5 %ID/g @ 24 h NP-CTRL (SD)	Gamma counter	(Xiao et al., 2012)
**Nano tripods**	20 nm	^{64}Cu	cRGD	Gold, PEG, Platinum	200 pmol/kg	U87MG xenografts in mice	150-200 mm^3	7.9 %ID/g Au-PEG-RGD, 2.6% ID/g Au-PEG, 3.8% ID/g blocked (SE)	PET	{Cheng, 2014 #190}
**Nanoemulsions**	120 nm	Aclacinomycin A (ACM)	Folate	DSPE-PEG2k, chol, vitamin E, pegylated	5 mg ACM/kg	KB xenografts in mice, folate deficient diet	~600 mm^3	0.1 %ID/g without folate, 0.2 %ID/g with folate	HPLC	(Ohguchi et al., 2008)

**Nanoparticles**	21.7 ± 0.07 nm	^{99m}Tc	Cyclic RGD	Gold	3.7 MBq	C6 xenografts in mice	0.1-0.3 g	3.48 ± 0.21 %ID/g @ 0.5h, 3.65 ± 0.19 %ID/g @ 1h, 2.49 ± 0.13 @ 3h, 1.94 ± 0.13 %ID/g @ 24h, 1.46±0.23 %ID/g @ 1h blocked (SD)	Well-type scintillation detector	(Morales-Avila et al., 2011)
**Nanoparticles**	85 ± 9 nm	^{111}In	Folate	PEG, gadolinium	10 mg/kg mouse	KB xenografts in mice	0.82 ± 0.2 g	5 %ID/tumor with folate @ 5 h, 7 %ID/tumor with folate @ 8 h, 4 %ID/organ without folate @ 5 hr, 9 %ID/organ without folate @ 8 hr (SD)	Gamma counter	(Oyewumi et al., 2004)
**Nanoparticles**	7 nm	Cy5+PEG+^{124}I	cRGD peptide	Silica, pegylated	20 µCi/mouse	M21 xenografts in mice	200 mm^3	1.5 %ID/g with RGDY, 1 %ID/g no RGDY @ 4 hr	Gamma counter	(Benezra et al., 2011)
**Nanoparticles**	30 nm	siRNA	EPPT peptide	PEG, iron oxide	10 mg/kg Fe	BT-20 xenografts mice	~0.5 cm in diameter	20 %ID/g	Gamma counter	(Kumar et al., 2010)
**Nanoparticles**	16 mm (TEM), 115 nm (DLS)	^{198}Au	BBN peptide	Gold	100 µL of 3 mg/mL	PC-3 xenografts in mice	~125 mg	0.48 %ID	Scintigraphic radio-counting methods	(Chanda et al., 2010)
**Nanoparticles**	80 nm		Transferrin	PEG, gold	4.5x10^{11} particles	neuro2A xenografts in mice		2-3 %ID (no difference with and without transferrin) @24h	ICP-MS	(Choi et al., 2010)
**Nanoparticles**	20, 200 nm	^{64}Cu, NIR dye	Aptamer to nucleolin	Silica, PEG	30 µCi	4T1 xenografts in mice		14.6 %ID/g NP-Apt, 6.2 %ID/g NP-nonspecific Ctrl (20nm)	PET, gamma counter	(Tang et al., 2012)
**Nanoparticles**	54.2 ± 1.9 nm with Ab	^{111}In	aHER2 (trastuzumab)	PEG, gold	10-12 MBq (=650-863 µg Au)	MDA-MB-361 xenografts in mice		1.23 ± 0.20 %ID/g, 2.20 ± 0.23 without Ab, 1.22 ± 0.27 %ID/g blocked @ 48h (SE)	Gamma counter, ICP-MS	(Chattopadhyay et al., 2012)
**Polymer nanocarriers**	10 nm	Dye (DY-670 or DY-676)	RGD and NGR peptides	p(HPMA-co-Ma-GG-DY-750-co-Ma-GG-AP)	2.5 nmol dye/mouse	BxPC3 (poorly leaky) and CT26 (highly leaky) xenografts in mice	~8 mm in diameter	~6 %ID/g CT26 @ 24, 48, 72 h ~3 %ID/g BxPC3 @24, 48, 72 h	FMT and micro-CT	(Kunjachan et al., 2014)
**Quantum Dots**	10-20 nm		RGD or RAD peptides	InAs/InP/ZnS QD, pegylated	200 pmol/mouse	U87MG xenografts in mice	200-500 mm^3	10.7 ± 1.5 %ID/g QD-RGD, 2.9 ± 0.3 %ID/g QD-PEG, 4.0 ± 0.5 %ID/g QD-RAD (SD)	IVIS Fluor (semi-quantitative)	(Gao et al., 2010)
**Shell Cross-linked Nanoparticles**	20 ± 3 nm	^{64}Cu	Folate	TETA-labeled folate functionalized PAA/PMA shell crosslinked nanoparticles + ^{64}Cu	370-440 kBq (3-5 mg/kg mouse)	KB xenografts in mice	10-100 mg for biodistribution, 0.3-0.6 g for autoradiography	3.4 ± 1.2 %ID/g with folate @ 10 min, 2.3 ± 0.7 %ID/g with folate @ 1 h, 5.9 ± 2.8 %ID/g with folate @ 4 h, 2.9 ± 3.1 %ID/g with folate @ 24 h, 2.2 ± 0.3 %ID/g without folate @ 10 min, 3.2 ± 0.7 %ID/g without folate @ 1 h, 6.0 ± 1.9 %ID/g without folate @ 4 h, 5.6 ± 0.9 %ID/g without folate @ 24 h (SD)	Gamma counter	(Rossin et al., 2005)

**SPIO micelles**	75 ± 11 nm	^{3}H	cRGD peptide	PEG-PLA copolymer micelle	6 mg Fe/kg mouse	A549 xenografts in mice	200-400 mm^3	1.3 ± 0.3 %ID/g cRGD-SPPM, 0.6 ± 0.3 %ID/g cRGD-free SPPM @ 1 hr (SD)	Liquid scintillation counter	(Khemtong et al., 2009)
**SPIONS**	10 nm bare SPIO, 68 ± 2 nm with cRGD	^{64}Cu, doxorubicin	Cyclo(RGDfC) peptides	SPIONS	5-10 MBq	U87MG xenografts in mice	6-8 mm in diameter ≈ 200 mm^3	5.4 ± 2.1 %ID/g cRGD-SPIO, 2.5%ID/g cRGD-free SPIO @ 24 hr (SD)	PET, gamma counter	(Yang et al., 2011)
**SPIONS**	20 nm	^{111}In	aChL6	Dextran, PEG	20-25 µCi	HBT 3477 xenografts in mice	100-350 mm^3	9 %ID/g, 0.5 %ID/g without Ab @ 48h	WBAR, gamma counter	(Natarajan et al., 2008)
